# Orthodontic incisor decompensation in orthognathic therapy—success and efficiency in three dimensions

**DOI:** 10.1007/s00784-020-03730-6

**Published:** 2020-12-11

**Authors:** Anja Quast, Petra Santander, Johanna Leding, Daniela Klenke, Norman Moser, Henning Schliephake, Philipp Meyer-Marcotty

**Affiliations:** 1 Department of Orthodontics, University Medical Center Goettingen, Robert-Koch-Str. 40, 37075 Goettingen, Germany; 2 Department of Oral and Maxillofacial Surgery, University Medical Center Goettingen, Robert-Koch-Str. 40, 37075 Goettingen, Germany

**Keywords:** Surgical-orthodontic treatment, Orthognathic surgery, Incisor inclination, Skeletal class, Cephalometry, Decompensation

## Abstract

**Objectives:**

Sufficient dental decompensation is crucial for treatment success in combined orthodontic-surgical treatment. The study’s objective was to determine the treatment success and efficiency in sagittal, vertical, and transversal decompensation.

**Methods:**

This longitudinal, observational study enrolled 52 adult patients, who underwent orthodontic-surgical treatment. Incisor inclinations and positions as well as skeletal changes were assessed pre-treatment (T1), pre-surgical (T2), and post-surgical (T3) by lateral cephalograms and CBCT scans.

**Results:**

Incisor decompensation was insufficient in all three dimensions. Sagittal: treatment efficiency did not differ between class II and III patients. Vertical: patients with open bite demonstrated pre-surgical bite deepening and insufficient surgical reduction of the maxillomandibular plane angle. Transversal: Dental midline deviations were not adapted to the skeletal asymmetry so that menton deviations were not properly corrected.

**Conclusions:**

Incisor decompensation was not as successful as requested in all three dimensions and the treatment ideal was seldom achieved.

**Clinical relevance:**

To improve the skeletal outcome, the orthodontist has to treat the patient with the desired surgical movements in mind and should critically evaluate the pre-surgical incisor decompensation before referral to the surgical team.

## Introduction

The comprehensive management of patients with severe malocclusions starts with a proper diagnosis and the decision whether the patient should be treated by interdisciplinary orthodontic-surgical treatment or by orthodontic therapy alone. The isolated orthodontic approach, known as camouflage, implies the dento-alveolar compensation of the skeletal anomaly with the consequence of non orthoaxial incisor inclination. In contrast, combined orthodontic-surgical treatment requires the complete dissolution of the natural dental compensation of the dysgnathic jaw relation in all three dimensions ending ideally in an orthoaxial incisor inclination [1].

Whether it is more advantageous to perform this orthodontic decompensation before or after the surgical intervention is still a matter of discussion [2, 3]. However, if the treatment team decides to perform orthodontics first, the orthodontist has to recognize existing dental compensations and should have absolute clarity which occlusal relationship is aimed for [4]. The pre-surgical occlusion dictates the surgical movements and, in the worst case, restricts them. This, not only leads to unstable results and longer postoperative treatment times but also to dissatisfaction of the patient and the treatment team [5].

Based on Proffit et al., successful and efficient treatment achieves normal occlusion, acceptable soft tissue, harmonic skeletal proportions, and dentofacial esthetics [6]. To quantify these properties, they distinguish between (1) treatment success, which means whether a measure achieved its ideal value (within an acceptable range), and (2) treatment efficiency, which is the percentage of change towards the ideal post-treatment goal. This kind of accurate assessment represents the true treatment dynamics and prevents that opposite changes in tooth positions, like proclination and reclination of anterior teeth, cancel each other out when summary statistics are applied [7].

Due to the great clinical impact of pre-surgical orthodontic treatment on the post-surgical result, the aim of this study was to evaluate the dental and skeletal changes during orthodontic-surgical treatment in all three dimensions using the method proposed by Proffit et al. [6]. While incisor decompensation in sagittal direction was assessed in a few studies [7–10], reports on vertical and transversal decompensation are completely missing in the literature. Furthermore, it has not yet been clarified whether pre-surgical orthodontic treatment is more efficient in class II or class III subjects [11]. Therefore, the purpose of this study was to determine treatment success and efficiency of sagittal, vertical, and transversal orthodontic decompensation in orthognathic therapy.

## Patients and methods

This longitudinal, observational study was approved by the institutional ethics committee (no. 7/1/16) in accordance with the Declaration of Helsinki. All patients gave their written informed consent to participate in the study.

### Patients

The study enrolled consecutively 52 healthy adult patients (Table 1) with severe malocclusions and indications for combined orthodontic-surgical treatment. All patients were categorized as > grade 4 according to the Index of Orthognathic Functional Treatment Need [12]. The following inclusion criteria were applied: (1) Surgery planning at our institution and (2) availability of high-quality pre-treatment lateral cephalograms and pre−/post-surgical cone-beam computed tomography (CBCT) images. Patients with cleft lip and/or palate or craniofacial syndromes were excluded from the study.

**Table 1:** Demographic and clinical characteristics of the study sample

Patient population	*N* = 52
male	*n* = 19
female	*n* = 33
Age at T1 (y)	*M* = 25.3; *SD* = 8.1
Age at T2 (y)	*M* = 27.4; *SD* = 7.9
Age at T3 (y)	*M* = 27.5; *SD* = 7.9
Skeletal classes at T1	
Skeletal class II (Wits > 2 mm)	*n = 26*
Skeletal class III (Wits < −2 mm)	*n = 26*
Vertical relation at T1	
hyperdivergent (MMPA > 26.5°)	*n = 23*
neutral (MMPA = [20.5; 26.5°])	*n = 16*
hypodivergent (MMPA < 20.5°)	*n = 13*
Asymmetry at T2	
Menton deviation < 2 mm Without isolated Le Fort I	*n =* 31 *n =* 28
Menton deviation ≥ 2 mm Without isolated Le Fort I	*n =* 21 *n =* 21
Surgical intervention	
Le Fort I and BSSO	*n* = 41
Isolated Le Fort I	*n* = 3
Isolated BSSO	*n = 8*

All patients were treated by the concept of orthodontics first. Orthodontic treatment was either performed by a referring orthodontist or at our clinic. Surgery planning was performed by a constant team (authors) of orthodontists and maxillofacial surgeons in the whole study period. All surgery procedures and pre−/post-surgical radiological controls were performed at our institution.

The sample size of 52 subjects (class II: *n* = 26; class III: *n* = 26) was determined in G*Power (v. 3.1.9.7, University of Düsseldorf) [13] applying a Mann-Whitney-U test for independent samples, with a significance level of 0.05, a power of 0.8, and an effect size of 0.825. The effect size was estimated based on the reported treatment efficiency of proclination of retroclined incisors (*M* = 150%), which is the main goal in class III subjects, versus the treatment efficiency of retroclination of proclined incisors (*M* = 51%), which is the aim in class II patients [7].

## Methods

Radiological data acquisition and cephalometric analysis were performed three times at T1 (2D lateral cephalogram) before orthodontic treatment, at T2 (3D CBCT) after orthodontic decompensation 4–6 weeks before orthognathic surgery, and at T3 (3D CBCT) within 2 weeks after orthognathic surgery.

At T1, the 2D cephalometric analysis was performed digitally using the software OnyxCeph^3^ (Image Instruments, Chemnitz, Germany). At T2 and T3 the cephalometric analysis was performed on the CBCT data. To get a good clinical comparability between T1 (lateral cephalograms) and T2/T3 (CBCT), a standardized procedure was implemented on the CBCT: the DICOM Data of the CBCT (Orange Dental PaX Zenith 3D, Biberach an der Riß, Germany; field of view 240 × 190 mm, voxel size 0,3 mm) were exported into the software Mimics InPrint 3.0, Edition Medical (Materialize, Leuven, Belgium), where the grayscale threshold was set between −10 and 800 for optimal bony representation. The maxilla and mandible were segmented, artifacts were eliminated, and the surface was automatically smoothed resulting in a 3D model. For better analysis at the dental level, dental plaster casts were scanned (S300 Ortho, Zirkonzahn S. R. L., Gais, Italy) and digitized. Using the software ProPlan CMF 3.0 (Materialize, Leuven, Belgium), the digitized dental casts were superimposed on the 3D model. To ensure accuracy in this procedure, this step was manually corrected if necessary. The resulting 3D model was then aligned to the Frankfurt Horizontal Plane (FH) and cephalometrically analyzed equally to the 2D lateral cephalogram [14].

The sagittal and vertical measurements were projected onto the midsagittal plane to receive 2D angles and distances. For transversal measurements, the distances were projected onto the frontal plane. The performed measurements at T1, T2, and T3 are listed in Table 2.

**Table 2:** Definition of sagittal, vertical, and transversal measurements at dental or skeletal levels. Sagittal and vertical parameters were measured on lateral cephalogram at T1 and were projected on the midsagittal plane at T2/T3 (CBCT cephalometry). Transversal measurements in the CBCT at T2 and T3 were projected on the frontal plane

Abbreviation	Measurement	Ideal (range)	Reference
Sagittal – dental			
U1-NA (°)	Inclination of the upper central incisors in relation to the NA-line	22 (19–25)	Steiner [29]
U1-NA (mm)	Bodily position of the upper central incisors in relation to the NA-line	4 (2–6)	Steiner [29]
U1-PP (°)	Inclination of the upper central incisors in relation to the maxillary plane	70 (65–75)	Rakosi [30]
L1-NB (°)	Inclination of the lower central incisors in relation to the NB-line	25 (22–28)	Steiner [29]
L1-NB (mm)	Bodily position of the lower central incisors in relation to the NB-line	4 (2–6)	Steiner [29]
L1-MP (°)	Inclination of the lower central incisors in relation to the mandibular plane	90 (87–93)	Rakosi [30]
Overjet (mm)	Shortest sagittal distance between the upper and lower central incisors	2 (1–4)	Proffit et al. [6]
Sagittal-skeletal			
Wits (mm)	Shortest distance between perpendiculars from points A and B onto the occlusal plane	0 (−2–2)	Jacobson [16]
Vertical-dental			
Overbite (mm)	Shortest vertical distance between the upper and lower central incisors	2 (1–4)	Proffit et al. [6]
Vertical-skeletal			
MMPA (°)	Maxillomandibular plane angle	23.5 (20.5–26.5)	Schwarz [17]
Transversal-dental			
L1-MSP	Shortest transversal distance between the contact point of the lower central incisors and the midsagittal plane	0 (−2–2)	Haraguchi et al. [27]
Transversal-skeletal			
Me-MSP	Shortest transversal distance between menton and the midsagittal plane	0 (−2–2)	Haraguchi et al. [27]

Treatment success and treatment efficiency were evaluated for each measurement as described previously [6, 7]. Treatment success was achieved when the measured value at T3 fell within the normal range for that measure. Treatment efficiency was calculated as $\frac{achieved\ tooth\ movement}{required\kern0.5em tooth\ movement}$ × 100. The closer this value was to 100%, the more efficient the treatment. Values higher than 100% indicated overcorrection, values between 0 and 100% meant undercorrection. Negative values were caused when the teeth were moved farther from ideal than initial.

### Statistics

Statistical analysis was performed in SPSS (v. 22, IBM, New York, USA). Intra- and interrater agreement of the measurements was assessed by Bland-Altman-plots [15] for lateral cephalograms and CBCTs separately. The data were assumed as non-normally distributed, and the median and interquartile range of the cephalometric measurements were reported at T1, T2, and T3.

To analyze the decompensation in the sagittal dimension, the patients were divided into skeletal class II and III according to the Wits appraisal [16]. Vertical decompensation was only investigated in hyperdivergent patients classified by the maxillomandibular plane angle [17]. Menton deviation ≥ 2 mm was used to discriminate between symmetric and asymmetric patients [18]. To evaluate the transversal decompensation, the absolute value of transversal dental (L1-MSP) and skeletal midline deviations (Me-MSP) from the midsagittal plane (MSP) was reported to prevent that deviations in opposite directions cancel each other out.

The treatment efficiency between class II and class III patients was compared by Mann-Whitney U test for independent samples. A subgroup analysis for patients with class II was performed based on incisor inclination by Kruskal-Wallis-test. The global level of significance was set at *p* < 0.05. Individual *p* values were adjusted by Bonferroni correction.

## Results

Incisor decompensation was investigated in sagittal, vertical, and transversal dimension in a sample of 52 patients. Dental and skeletal positions were assessed by cephalometric measurements in 156 lateral cephalograms or CBCT scans at three time points. Bland-Altman plots revealed high intra- and interrater agreement for these measurements with average differences < 0.5 mm/0.5° and small limits of agreement.

### Sagittal decompensation

The sagittal dental and skeletal measurements at T1, T2, and T3 are reported in Table 3. As expected, class II patients had slightly proclined upper and lower incisors in protruded positions at T1, which were orthodontically retroclined in the upper but not the lower jaw from T1 to T2. The treatment success for the three upper dental variables was improved (U1-NAmm: +7.7%; U1-PP: +7.7%) or equal (U1-NA°) from T2 to T3. In contrast, the treatment success for the lower incisors was only improved for one investigated variable (L1-MP: +3.9%), whereas the other variables were impaired (L1-NB° -11.6%) or were equal (L1-NBmm) from T2 to T3. The sagittal jaw relation was overcorrected by surgery, which is revealed by the negative Wits appraisal at T3. 12 of 26 patients (46.2%) were postoperatively classified as skeletal class I, 9 patients (34.6%) were overcorrected in class III and 5 patients (19.2%) kept their class II.

**Table 3:** Median and interquartile range of sagittal dental and skeletal measurements at T1 (pre-treatment), T2 (pre-surgical) and T3 (post-surgical). If the value of a measure fell within the normal range for that variable, the treatment was rated “successful”

Measurement	Reference Ideal (range)	T1 *Median* *(IQR)*	T2 *Median* *(IQR)*	T3 *Median* *(IQR)*	Treatment success at T2 *n* (%)	Treatment success at T3 *n* (%)
Class II (*n* = 26)						
U1-NA (°)	22 (19–25)	25.8 (14.3)	18.5 (9.7)	15.2 (9.8)	7 (26.9)	7 (26.9)
U1-NA (mm)	4 (2–6)	6.3 (5.8)	2.4 (4.6)	2.2 (4.4)	11 (42.3)	13 (50)
U1-PP (°)	70 (65–75)	66.1 (17.7)	73.3 (9.6)	73 (10.2)	10 (38.5)	12 (46.2)
L1-NB (°)	25 (22–28)	26.2 (9.3)	25.9 (8.5)	29 (11.1)	10 (38.5)	7 (26.9)
L1-NB (mm)	4 (2–6)	5.7 (4.7)	5.5 (4.1)	6.3 (4)	13 (50)	13 (50)
L1-MP (°)	90 (87–93)	96.4 (11)	97.8 (11)	98.7 (14.4)	5 (19.2)	6 (23.1)
Overjet (mm)	2 (1–4)	8.8 (4.8)	9 (2.4)	2.9 (1.2)	–	21 (80.8)
Wits (mm)	0 (−2–2)	4 (3.1)	5.3 (4.3)	−1.4 (4.1)	–	12 (46.2)
Class III (*n* = 26)						
U1-NA (°)	22 (19–25)	30.2 (15.9)	26.6 (12.2)	20 (9.4)	4 (15.4)	9 (34.6)
U1-NA (mm)	4 (2–6)	5.9 (4.9)	5.6 (2.1)	3.4 (3.2)	13 (50)	15 (57.7)
U1-PP (°)	70 (65–75)	62.9 (11.3)	64.3 (15)	65.6 (9.6)	8 (30.8)	9 (34.6)
L1-NB (°)	25 (22–28)	22.1 (8.6)	25.4 (8.5)	22.3 (9.3)	14 (53.8)	6 (23.1)
L1-NB (mm)	4 (2–6)	3.9 (3.9)	5 (3.2)	3.9 (2.7)	18 (69.2)	20 (76.9)
L1-MP (°)	90 (87–93)	82.8 (12.6)	91.2 (12.2)	90.2 (14.7)	7 (26.9)	9 (34.6)
Overjet (mm)	2 (1–4)	−4.4 (7.1)	−2.9 (4.3)	3.2 (1.5)	–	20 (76.9)
Wits (mm)	0 (−2–2)	−9.2 (7.5)	−7.3 (7.6)	−3.3 (3.4)	–	9 (34.6)

The class III subjects initially demonstrated proclined upper incisors in a protruded position, which were retroclined from T1 to T2. The lower incisors were retroclined in an ideal position at T1 and were proclined during orthodontic decompensation. Additionally, the upper and lower incisor inclinations and positions changed by surgical jaw movements from T2 to T3. Upper and lower incisors achieved more often a correct bodily position than a correct inclination in class III patients.

Treatment success increased from T2 to T3 in all variables (U1-NA°: +19.2%; U1-NAmm: +7.7%; U1-PP: +3.8%; L1-NBmm: +8.7%; L1-MP: 7.7%) except for the lower incisor inclination in relation to the cranial base (L1-NB -30.7%).

Following surgery, at T3 the overjet and the Wits appraisal were normalized, even though only 9 of 26 patients (34.6%) were successfully treated into skeletal class I, while 17 of 26 patients (65.4%) maintained a skeletal class III relation.

Treatment efficiency differed significantly between class II and class III subjects for surgical changes but not for orthodontic incisor decompensation (Table 4). The skeletal jaw discrepancy was more efficiently corrected in class II cases than in class III patients.

**Table 4:** Comparison of treatment efficiency in sagittal decompensation from T1 (pre-treatment) to T2 (pre-surgical) and from T2 to T3 (post-surgical) between class II and III patients. α-levels were adjusted by Bonferroni correction

	Treatment efficiency (%)		
Measurement	Class II (*n* = 26) *Median (IQR)*	Class III (*n* = 26) *Median (IQR)*	*p* value
From T1 to T2: orthodontic changes			
U1-NA °	116 (117)	37 (88)	.52
U1-NA mm	64 (142)	50 (146)	1
U1-PP	62 (104)	3 (115)	.403
L1-NB °	50 (166)	73 (102)	1
L1-NB mm	29 (118)	45 (158)	1
L1-MP	0 (99)	38 (41)	.286
From T2 to T3: surgical changes			
Overjet	86 (18)	118 (34)	< .001*
Wits	85 (85)	57 (38)	.013*

Treatment efficiency: 0–100% = treatment towards the ideal

Treatment efficiency: > 100% = overcorrection

(* = significant)

While the overjet was often overcorrected in class III subjects, no class II patient overshoot the ideal (Table 5). The majority of patients demonstrated an orthodontic correction towards the ideal incisor inclination/position and was often overcorrected beyond ideal. The highest percentage of overcorrection was observed in class II patients for the upper incisor inclination (U1-NA°). However, subgroup analysis revealed that the initial incisor inclination, i.e., Class II division 1 or 2, did not affect this overcorrection (Table 6).

**Table 5:** Treatment direction in sagittal decompensation from T1 (pre-treatment) to T2 (pre-surgical) and from T2 to T3 (post-surgical) for patients with class II (*n* = 26) and III (*n* = 26)

Measurement	Towards ideal *n (%)*	Away from ideal *n (%)*	Overcorrection *n* (%)
Class II (*n* = 26) From T1 to T2: orthodontic changes			
U1-NA °	8 (30.8%)	4 (15.4%)	14 (53.8%)
U1-NA mm	10 (38.5%)	6 (23.1%)	10 (38.5%)
U1-PP	11 (42.3%)	5 (19.2%)	10 (38.5%)
L1-NB °	10 (38.5%)	7 (26.9%)	9 (34.6%)
L1-NB mm	11 (42.3%)	10 (38.5%)	5 (19.2%)
L1-MP	9 (34.6%)	12 (46.2%)	5 (19.2%)
From T2 to T3: surgical changes			
Overjet	26 (100%)	0 (0%)	0 (0%)
Wits	12 (46.2%)	2 (7.7%)	12 (46.2%)
Class III (*n* = 26) From T1 to T2: orthodontic changes			
U1-NA (°)	14 (53.8%)	7 (26.9%)	5 (19.2%)
U1-NA (mm)	12 (46.2%)	7 (26.9%)	7 (26.9%)
U1-PP (°)	9 (34.6%)	13 (50%)	4 (15.4%)
L1-NB (°)	13 (50%)	5 (19.2%)	8 (30.8%)
L1-NB (mm)	10 (38.5%)	10 (38.5%)	6 (23.1%)
L1-MP (°)	23 (88.5%)	1 (3.8%)	2 (7.7%)
From T2 to T3: surgical changes			
Overjet (mm)	4 (15.4%)	1 (3.8%)	21 (80.8%)
Wits (mm)	21 (80.8%)	1 (3.8%)	4 (15.4%)

**Table 6:** Comparison of treatment efficiency in sagittal decompensation from T1 (pre-treatment) to T2 (pre-surgical) and from T2 to T3 (post-surgical) between retro-, normo- and proclined incisors in class II patients. α-levels were adjusted by Bonferroni correction

	Treatment efficiency (%)			
Measurement	Retroclined incisors (*n* = 7) *Median* *(IQR)*	Normoinclined incisors (*n* = 6) *Median* *(IQR)*	Proclined incisors (*n* = 13) *Median* *(IQR)*	*p* value
From T1 to T2: orthodontic changes				
U1-NA °	40 (85)	144 (1114)	116 (138)	.73
U1-NA mm	10 (126)	51 (398)	133 (107)	1
U1-PP	41 (87)	11 (683)	107 (116)	1
From T2 to T3: surgical changes				
Overjet	75 (28)	87 (35)	86 (14)	1
Wits	78 (87)	118 (200)	86 (82)	1

Treatment efficiency: 0–100% = treatment towards the ideal

Treatment efficiency: > 100% = overcorrection

### Vertical decompensation

The vertical decompensation was analyzed in 23 hyperdivergent patients (Table 7). Against the treatment plan, anterior open bite closure was observed pre-surgically from T1 to T2 in many cases, and the overbite was even overcorrected in 3 patients (Table 8). At T3, 18 of 23 patients (78.3%) still had a hyperdivergent skeletal relation regarding MMPA. The rest achieved the ideal value or was overcorrected. Overall, dental correction of the open bite was more successful compared with the skeletal correction.

**Table 7:** Median, minimum, and maximum of vertical dental and skeletal measurements at T1 (pre-treatment), T2 (pre-surgical) and T3 (post-surgical) for hyperdivergent patients (*n* = 23). If at T3 the value of a measure fell within the normal range for that measure, the treatment was rated “successful”

Measurement	Reference Ideal (range)	T1 *Median* *(IQR)*	T2 *Median* *(IQR)*	T3 *Median* *(IQR)*	Treatment success at T3 *n* (%)
Overbite (mm)	2 (1–4)	−1.2 (4.3)	0.5 (2.1)	1.6 (1.4)	16 (69.6)
MMPA (°)	23.5 (20.5–26.5)	34.1 (7.3)	34.8 (5.1)	29 (6.0)	3 (13)

**Table 8:** Treatment direction in vertical decompensation from T1 (pre-treatment) to T2 (pre-surgical) and from T2 to T3 (post-surgical) for hyperdivergent patients (*n* = 23)

Measurement	Treatment efficiency (%) *Median (IQR)*	Towards ideal *n (%)*	Away from ideal *n (%)*	Overcorrection *n* (%)
From T1 to T2				
Overbite	55 (94)	14 (60.9%)	6 (26.1%)	3 (13%)
MMPA	5 (28)	16 (69.6%)	7 (30.4%)	0 (0%)
From T2 to T3				
Overbite	77 (91)	15 (65.2%)	2 (8.7%)	6 (26.1%)
MMPA	56 (54)	16 (69.6%)	2 (8,7%)	5 (21.7%)

Treatment efficiency: 0–100% = treatment towards the ideal

Treatment efficiency: > 100% = overcorrection

### Transversal decompensation

Our evaluation of transversal decompensation focused on asymmetries in the lower jaw. Patients with isolated Le Fort I surgeries had no surgical changes in the lower jaw and were excluded from these analyses. Therefore, the transversal decompensation was evaluated in 49 patients divided in two groups—symmetric versus asymmetric patients. Dental midline correction was more successful than skeletal midline correction in both groups (Table 9). Treatment success was higher for symmetric than asymmetric subjects, especially for menton deviation. Treatment efficiency ranged widely in symmetric patients and overcorrection or treatment away from the ideal was observed frequently (Table 10).

**Table 9:** Median, minimum, and maximum of the absolute value of transversal dental (L1-MSP) and skeletal midline deviations (Me-MSP) and their discrepancy (L1-Me) at T2 (pre-surgical) and T3 (post-surgical) for symmetric and asymmetric patients. If at T3 the value of a measure fell within the normal range for that measure, the treatment was rated “successful”

Measurement	Reference Ideal (range)	T2 *Median* *(IQR)*	T3 *Median* *(IQR)*	Treatment success at T3 *n* (%)
Symmetric (*n* = 28)				
L1-MSP (mm)	0 (0–2)	0.9 (1.1)	0.9 (1.5)	22 (79%)
Me-MSP (mm)	0 (0–2)	0.6 (0.7)	1.5 (2)	20 (71%)
Me-L1 (mm)	0 (0–2)	0.9 (1.1)	1.3 (1.3)	23 (82%)
Asymmetric (*n* = 21)				
L1-MSP (mm)	0 (0–2)	2.8 (3.1)	1.5 (1.7)	14 (67%)
Me-MSP (mm)	0 (0–2)	5 (3.7)	3.2 (2.2)	4 (19%)
Me-L1 (mm)	0 (0–2)	1.9 (2.8)	1.5 (1.6)	12 (57%)

**Table 10:** Treatment efficiency and direction in transversal decompensation from T1 (pre-treatment) to T2 (pre-surgical) and from T2 to T3 (post-surgical) for symmetric (*n* = 28) and asymmetric patients (*n* = 21)

	From T2 to T3			
Measurement	Treatment efficiency (%) *Median (IQR)*	Towards ideal *n (%)*	Away from ideal *n (%)*	Overcorrection *n* (%)
Symmetric (*n* = 28)				
L1-MSP	65 (143)	11 (39%)	7 (25%)	10 (36%)
Me-MSP	31 (525)	3 (11%)	12 (43%)	13 (46%)
Asymmetric (*n* = 21)				
L1-MSP	29 (81)	14 (67%)	6 (29%)	1 (4.8%)
Me-MSP	32 (56)	15 (71%)	5 (24%)	1 (4.8%)

Treatment efficiency: 0–100% = treatment towards the ideal

Treatment efficiency: > 100% = overcorrection

## Discussion

This longitudinal, cephalometric study provided an entire overview of the pre-surgical orthodontic decompensation in all three dimensions—sagittal, vertical, and transversal. A distinctive patient cohort characterized by severe malocclusion could be analyzed form the beginning of treatment to the time point of surgical intervention. The main outcome was that in the pre-surgical treatment phase an ideal orthodontic position of the upper and lower incisors was difficult to achieve. The incisor decompensation was not as successful as requested and the treatment ideal was seldom achieved. However, the purpose of this study was not to criticize orthodontic-surgical treatment in general but to emphasize the problems facing the orthodontists and surgeons. Moreover, it highlights the effect of pre-surgical orthodontic treatment on the overall surgical result.

For the examination of dental and skeletal outcomes, lateral cephalograms (at T1) and CBCT scans (at T2 and T3) were available, which offered us the possibility to evaluate the change of incisor positions in three dimensions directly before and after surgery. In the last decades, measurements in CBCTs gained wide acceptance and proofed to be at least as accurate as measurements in lateral cephalograms [19, 20]. However, cephalograms are still the standard of care in orthodontic treatment planning, and according to the ALARA principles, there is no need to perform a CBCT in the pre-orthodontic treatment stage by default [21]. Moreover, 2D conventional and 3D CBCT analyses of standardized linear and angular cephalometric variables, as used in this study, are assumed as clinically comparable [22]. Therefore, no CBCT scans were used at T1 and transversal decompensation was only assessable at T2 and T3.

The CBCT scans at T3 were taken within 2 weeks after surgery so that the post-surgical orthodontic treatment phase was not assessed by this study. During post-surgical treatment, an improvement of the dental occlusion is usually observed. Further changes in incisor inclination and position may be achieved by the use of intermaxillary elastics or approximal enamel reduction. However, the post-surgical treatment phase also coincides with skeletal relapse. Thus, the prompt evaluation of surgical-induced changes of the skeletal and dental variables had the advantage to avoid any falsification of our results.

Since the patients in our study were treated by a range of clinicians in private practices and an orthodontic clinic, one might argument that treatment modalities and different levels in clinical experience have affected the outcome. However, this study design mirrors the real clinical practice [9] and reflects the situation the planning team of surgeons and orthodontist has to cope with. Furthermore, Potts et al. reported no differences in treatment efficiency between experienced and novice clinicians [8]. Therefore, the presented results are relevant to all practicing orthodontists.

### Sagittal decompensation

In consistence with previous studies, sagittal incisor decompensation was not as successful as requested and treatment efficiency ranged widely from treatment away from the ideal to overcorrection [6–8]. As proclination of retroclined lower incisors is easier than retroclination of proclined incisors, we expected a more efficient orthodontic treatment in class III patients compared with class II patients for the lower jaw. But no significant difference in orthodontic treatment efficiency between class II and III patients was observed. Instead, it was the surgical correction of skeletal jaw discrepancies that was more efficient in class II cases than in class III patients. To avoid any selection bias, the patients in our study were included consecutively without considering the severity of their malocclusion. However, the patients with skeletal class III demonstrated more severe skeletal discrepancies pre-surgical according to the Wits appraisal compared with the class II patients, which might cause the difference in treatment efficiency.

Interestingly, a physiological overjet was more often achieved than a neutral jaw relation, which indicates that the success in overjet correction was based on a combination of dental compensation and skeletal changes. This seems to be a common problem in surgical-orthodontic treatments [7–9, 23, 24] and limits the surgical improvement of facial proportions. Keeping this in mind, the orthodontist should try to decompensate the incisors as much as possible within the physiological limits. Furthermore, the surgical movements affect the incisor inclinations and positions. Clockwise rotation of the maxilla, for example, leads to retroclination of the upper incisors in relation to the anterior cranial base (OK1-NA°). Therefore, the surgical movements should be determined prior to pre-surgical orthodontics in close interdisciplinary communication with the surgeon. This is not only important to improve the treatment result but also shortens treatment time [25].

Treatment success of lower incisor inclination in class II patients demonstrated divergent results for the surgical-induced inclination changes in relation to the anterior cranial base (U1-NB°) and the mandibular plane (L1-MP) raising the question which variable is more reliable during pre-surgical decompensation to achieve the best surgical result. As maxillary and mandibular translations and rotation by surgery may alter the incisor inclination in relation to the cranial base (U1-NA° and L1-NB°) more than in relation to the maxillary or mandibular plane (U1-PP and L1-MP), we recommend to focus on the latter.

### Vertical decompensation

Vertical decompensation was solely assessed for patients with open, but not deep bite due to varying treatment strategies in this type of malocclusion. Deep bites can be either treated pre-surgical by intrusion of the incisors or post-surgical by leveling of the curve of Spee. The decision which option should be favored is complex and depends on the height of the lower face and on the need of surgical mandibular advancement. In hyperdivergent patients with open bite, the treatment strategy is clearer, and the aim is to decrease the overbite pre-surgically. Against this goal, we observed an increase of overbite from T1 to T2 in this study, which affected the surgical correction of the maxillomandibular plane angle. Since straight wire appliances induce anterior bite deepening in the early stages of treatment [26], this finding is not surprising. As a clinical consequence, the orthodontist should consider treating these patients with segmented arch wires to retain or even increase the anterior open bite pre-surgically.

### Transversal decompensation

Perfect transversal decompensation means that the menton deviation and the deviation of the dental midline in the lower jaw should be congruent to each other pre-surgically. Otherwise the surgical correction of the dental midline will cause unwanted menton deviation. Due to insufficient transversal decompensation, we observed this effect in symmetric and asymmetric patients in the present study.

However, the herein used threshold of 2 mm was chosen pretty strict to detect also small discrepancies in accordance with previous research [27]. As discriminative thresholds of chin asymmetry up to 6 mm have been reported and orthodontists accepted dental midline deviations of 4 mm before they downgraded attractiveness [28], the observed non-ideal transversal decompensations might be clinically tolerable. Nevertheless, it should be avoided to correct dental problems surgically. A precise orthodontic treatment plan with evaluation of the dental and skeletal midline helps to improve the pre-surgical transversal decompensation.

## Conclusions

Within the limitations of this study, the results underpin that an individual diagnosis and treatment planning are of great importance for the success of combined orthodontic-surgical therapy. Keeping the following conclusion in mind may help to improve the personal result:

1. Incisor decompensation is often insufficient in all three dimensions—sagittal, vertical, and transversal.
2. Surgical movements affect incisor inclination and should be already considered during the pre-surgical orthodontic sagittal decompensation.
3. Treatment efficiency of sagittal incisor decompensation does not differ between class II and III patients.
4. Patients with open bite frequently demonstrate pre-surgical bite deepening which might explain the less successful surgical reduction of the maxillomandibular plane angle.
5. If dental and skeletal midline deviations are not adapted to each other, they cause insufficient correction of menton deviations.

## Abbreviations

2D/3D: Two- /three-dimensional
CBCT: Cone-beam computed tomography
FH: Frankfurt Horizontal plane
MSP: Midsagittal plane
MMPA: Maxillomandibular plane angle
